# Investigating the Impact of Hearing Aid Use and Auditory Training on Cognition, Depressive Symptoms, and Social Interaction in Adults With Hearing Loss: Protocol for a Crossover Trial

**DOI:** 10.2196/resprot.8936

**Published:** 2018-03-23

**Authors:** Joanna Nkyekyer, Denny Meyer, Peter J Blamey, Andrew Pipingas, Sunil Bhar

**Affiliations:** ^1^ Australian Research Council Training Centre in Biodevices Faculty of Science, Engineering and Technology Swinburne University of Technology Hawthorn Victoria Australia; ^2^ Department of Statistics, Data Science and Epidemiology Swinburne University of Technology Hawthorn Victoria Australia; ^3^ Blamey and Saunders Hearing Pty Ltd Melbourne Australia; ^4^ Centre for Human Psychopharmacology Swinburne University of Technology Hawthorn Victoria Australia; ^5^ Department of Psychological Sciences Swinburne University of Technology Hawthorn Victoria Australia

**Keywords:** sensorineural hearing loss, hearing aids, crossover design

## Abstract

**Background:**

Sensorineural hearing loss is the most common sensory deficit among older adults. Some of the psychosocial consequences of this condition include difficulty in understanding speech, depression, and social isolation. Studies have shown that older adults with hearing loss show some age-related cognitive decline. Hearing aids have been proven as successful interventions to alleviate sensorineural hearing loss. In addition to hearing aid use, the positive effects of auditory training—formal listening activities designed to optimize speech perception—are now being documented among adults with hearing loss who use hearing aids, especially new hearing aid users. Auditory training has also been shown to produce prolonged cognitive performance improvements. However, there is still little evidence to support the benefits of simultaneous hearing aid use and individualized face-to-face auditory training on cognitive performance in adults with hearing loss.

**Objective:**

This study will investigate whether using hearing aids for the first time will improve the impact of individualized face-to-face auditory training on cognition, depression, and social interaction for adults with sensorineural hearing loss. The rationale for this study is based on the hypothesis that, in adults with sensorineural hearing loss, using hearing aids for the first time in combination with individualized face-to-face auditory training will be more effective for improving cognition, depressive symptoms, and social interaction rather than auditory training on its own.

**Methods:**

This is a crossover trial targeting 40 men and women between 50 and 90 years of age with either mild or moderate symmetric sensorineural hearing loss. Consented, willing participants will be recruited from either an independent living accommodation or via a community database to undergo a 6-month intensive face-to-face auditory training program (active control). Participants will be assigned in random order to receive hearing aid (intervention) for either the first 3 or last 3 months of the 6-month auditory training program. Each participant will be tested at baseline, 3, and 6 months using a neuropsychological battery of computer-based cognitive assessments, together with a depression symptom instrument and a social interaction measure. The primary outcome will be cognitive performance with regard to spatial working memory. Secondary outcome measures include other cognition performance measures, depressive symptoms, social interaction, and hearing satisfaction.

**Results:**

Data analysis is currently under way and the first results are expected to be submitted for publication in June 2018.

**Conclusions:**

Results from the study will inform strategies for aural rehabilitation, hearing aid delivery, and future hearing loss intervention trials.

**Trial Registration:**

ClinicalTrials.gov NCT03112850; https://clinicaltrials.gov/ct2/show/NCT03112850 (Archived by WebCite at http://www.webcitation.org/6xz12fD0B).

## Introduction

### Background and Rationale

Hearing loss is a common experience for older adults and is one of the leading causes of nonfatal disease burden for Australians aged 65 years and older [1,2]. Sensorineural hearing loss or presbycusis is the most prevalent hearing-related chronic condition affecting this population; however, it is often underdetected and undertreated. This type of hearing loss cannot be medically or surgically treated [3-6]. The number of adults who suffer from sensorineural hearing loss worldwide is likely to increase rapidly as the population ages [7].

Recent studies have reported that hearing loss among older adults is strongly and independently associated with accelerated cognitive decline [8-13]. Epidemiologic and longitudinal studies have demonstrated that older people aged between 70 and 79 years with hearing impairment, who live in the community, have a 24% increased risk of a decline in cognitive function and may experience a 30% to 40% higher rate of cognitive decline over a 6-year period than those without hearing loss [9,14,15]. The proposed theories to explain the above association relate to the effects of hearing loss on cognitive load and cognition reserve, and the effects of hearing impairment on brain structure and shared pathologic etiology, social isolation, and depressive symptoms [13]. Social isolation and communication impairments caused by hearing loss are known to lead to loneliness and depression in older adults [16,17], often resulting in a negative perception of one’s own health and a decline in daily activities, with associated declines in cognitive performance.

In aural rehabilitation, hearing aid use and auditory training strategies contribute to improving auditory abilities. The basic function of hearing aids is acoustic amplification of sound signals with the aim of restoring the audibility of sounds, thus helping to improve speech perception [18]. Studies have examined the effects of hearing aid use by older adults on a broad range of cognitive functions, such as information-processing speed, memory, and verbal fluency. Preliminary research evidence has suggested that hearing aids may improve the cognitive abilities, social, emotional, psychological, and physical well-being of people [18-21]. Some studies reporting the cognitive and psychological benefits of using hearing aids in elderly people have shown that the effects of hearing aid use are most distinctive in the early periods of use [6]. Despite the high prevalence of hearing loss in older adults, and the consequences for health outcomes, people are generally slow to acquire hearing aids [22]. Less than 25% of people who would benefit from hearing aids actually own them [23]. Existing research in this area, attempting to describe the effects of hearing aids on cognition, often assessed global mental status rather than cognitive performance and often examined only a single measure of hearing [6,19,24], thus limiting the insights gained. These studies also lack data on the duration of hearing impairment and loosely define hearing aid use as the self-reported use of a hearing aid in either or both ears, thus making it unclear about how hearing loss may affect performance on measures of cognition.

Auditory training is the use of instruction, drill, or practice, designed to increase the amount of information that hearing contributes to a person’s total perception [25]. For example, a person with a hearing impairment who is fitted with a new hearing aid may benefit from instruction and practice in recognizing sounds through the aid. Research has shown that new hearing aid users show greater benefit from auditory training than experienced hearing aid users [26]. Auditory training also shares processes in common with cognitive training for improving working memory, attention, and communication. Studies have shown that auditory training can produce prolonged cognitive performance improvements [27,28] and improve speech understanding [29,30]. Other studies have shown that the benefits of training for people with hearing loss in terms of improved speech understanding are best achieved if an integrated auditory-cognitive training approach is adopted [31].

Although the concept of auditory training is not new, its popularity has declined in recent years, and only a small proportion of audiologists (fewer than 10%) offer auditory training to patients with hearing impairment [32]. Also, limited auditory training effort has been directed toward adults with impaired hearing, and the focus of auditory training has historically been directed toward young children with profound or severe to profound hearing loss [33,34].

Studies have investigated the effects of auditory training with laptops and computers, such as with the Listening and Communication Enhancement (LACE) software, on generalization to speech perception, self-report of communication difficulties, and cognition [27,28,33]. The results of these studies have often demonstrated the efficacy of auditory training, despite the computerized method of auditory training perhaps resulting in lower compliance with training protocols [32]. In addition, Saunders et al [35] found that LACE training did not result in improved outcomes over a standard-care hearing aid intervention on its own. Furthermore, according to research studies [36,37], there are still a large number of outstanding questions on the benefits of auditory training, such as which aspects of auditory training protocols contribute to learning, how auditory training generalizes to benefits in everyday communication, how individual characteristics interact with training outcomes to identify candidacy for auditory training, and the identification of outcome measures that are appropriate and sufficiently sensitive.

Research has shown that hearing aid devices alone do not always adequately compensate for sensory losses despite significant technological advances in digital technology [38]. Therefore, the focus of intervention will consider face-to-face auditory training in conjunction with a hearing aid device, whereas the comparator (control) group will consider individualized face-to-face auditory training on its own.

### Study Objective

Extending upon preliminary findings [27,28,36], the objective of this study is to investigate whether wearing hearing aids will improve the impact of individualized face-to-face auditory training on cognition, depression, and social interaction for adults with sensorineural hearing loss in a crossover intervention trial.

The study is based on the following hypotheses:

1. In adults with sensorineural hearing loss, hearing aids in combination with face-to-face auditory training will be more efficient for improving cognition than face-to-face auditory training on its own.
2. In adults with sensorineural hearing loss, hearing aids in combination with face-to-face auditory training will be more efficient for improving depression and social interaction than face-to-face auditory training on its own.

## Methods

### Trial Design

This study has a randomized crossover trial design. It attained ethics approval on July 22, 2016 (Swinburne’s Human Research Ethics Committee protocol number SHR Project 2016/159).

All participants will undergo an individualized face-to-face auditory training program for a period of 6 months and will be randomly allocated to one of the following groups:

1. Participants who will be fitted with hearing aids only for the first 3 months of the auditory training program—*Group A.*
2. Participants who will be fitted with hearing aids only for the last 3 months of the auditory training program—*Group B.*

Participants will be tested at baseline, and at 3 and 6 months in terms of cognition, depressive symptoms, social interaction, and hearing satisfaction.

A crossover design is chosen to allow each participant to serve as their own control [39]. Group A participants will have the option to withdraw from the study after 3 months if they decide to purchase hearing aids immediately. Similarly, group B participants will also have the option of withdrawing from the study at any time. As all participants will receive auditory training for the entire duration of the study to address their hearing loss, participants will benefit from the study even when the hearing aid intervention is not in place.

### Study Setting

This study is set in Melbourne, Australia. The study will recruit men and women who are living independently—both in supported independent living accommodation and living independently in the community.

### Eligibility Criteria

To be eligible to participate in the study, participants must satisfy all of the following criteria:

1. Be aged between 50 and 90 years
2. Have a good working knowledge of English
3. Have mild (26-40 dB) or moderate (41-70 dB) symmetric sensorineural hearing loss with a pure-tone average threshold of 0.5 to 4 kHz in both ears
4. Have never worn hearing aids previously
5. Be willing to wear hearing aids for 3 months
6. Be willing to undergo weekly auditory training for a period of 6 months
7. Be willing to provide written consent to participate in the study

### Exclusion Criteria

Participants will be unable to participate in the study if they have any significant visual impairment that would prevent reading or performing computer-based tasks requiring color recognition. Additionally, study participants with severe or profound hearing loss will not be eligible to take part in the study. Finally, participants with suspected cognitive impairment (defined as a score ≤24 on the Mini-Mental State Examination [MMSE]) will be excluded.

### Intervention

#### Fitting of Hearing Aids for Group A and Group B Participants

Participants will be loaned and fitted with 2 Blamey Saunders hearing aids known as LOF (LOF is the current trade name used by the manufacturer for the model of hearing aid in this study. The name LOF was derived from its original name, Liberty Open-Fit). The hearing aids will be fitted to participants according to the Blamey and Saunders protocol and using the prescription procedures from the National Acoustics Laboratories (NAL) protocol for fitting hearing aids as a guide [40]. Explanation of the hearing aid usage, insertion of the aids and batteries, along with a step-by-step guide on how to use the hearing aid will also be provided. To increase hearing aid compliance, support will be provided post fitting (after 1 month) to make sure that each participant is progressing with his or her hearing aid. Counseling and other compliance-improving policies [41-43] will be provided when participants receive their new hearing aids and at their postfitting appointment. An automatic internet-based data logging function installed in the hearing aids will be used to assess hours of hearing aid use.

#### Auditory Training

Historically, auditory training has been provided in a face-to-face setting that centered on a range of auditory skills, including detection, discrimination, identification, and comprehension. Training often incorporated both drill-like activities, described as analytic therapy activities, and paragraph comprehension activities, which were synthetic in nature. For both activities, the auditory skills that were trained used various stimuli such as syllables, words, phrases, sentences, and continuous discourse [38].

All participants enrolled into the study will undergo weekly individualized face-to-face auditory training for a period of 6 months. Over the 6-month period, each participant will participate in two 12-week individualized speech tracking programs. Participants living in supported independent living accommodation will attend their auditory training sessions at their place of residence, once per week for the 6-month period. Participants living independently in the community will attend their auditory training sessions once per week at Swinburne University of Technology. Each auditory training session will last for approximately 15 min.

The type of counseling intervention that will be provided to participants is called Continuous Discourse Speech Tracking [44]. A key aspect of this approach is that the training will involve interaction (a vital component of real-life communication) between the researcher and the participants. In this process, the researcher will articulate a sentence or phrase in a novel or short story, and the task of the participant will be to repeat back verbatim the sentence or phrase. If the repetition is correct, the researcher will articulate the next phrase or sentence. If the repetition is incorrect, the researcher will repeat the phrase or sentence, or a portion of it, or may use other repair strategies, until the sentence or phrase is correctly repeated in its entirety. The procedure will be timed for 15 min and scored in number of words per minute transmitted. Tracking rate will be calculated as the number of words correctly repeated divided by the time elapsed.

This program is adopted for this sample population because training materials could be tailored to the personal interests of participants. The materials chosen for the speech tracking program will consist of short stories, which will be long enough to last for a full 12-week program. A new story will be started at the beginning of each 12-week program.

### Outcome Measures

The primary outcome measure will be changes in cognitive performance as measured by the spatial working memory component of the Swinburne University Computerized Cognitive Assessment Battery (SUCCAB). Reliability and validity assessment has demonstrated that the SUCCAB, especially the spatial working memory component of this battery, is sensitive to aging and intervention and correlates strongly with memory subsets in the Wechsler Adult Intelligence Scale –Fourth Edition [45-47].

Secondary measures include the other SUCCAB cognition measures, social interaction measured using the Berkman-Syme Social Network Index, and depressive symptoms measured using the Geriatric Depression Scale (GDS). Hearing satisfaction (with or without hearing aids) will be measured using the Abbreviated Profile of Hearing Aid Benefit (APHAB) Inventory.

All outcomes will be measured at baseline, after 3 months, and after 6 months (Table 1).

**Table 1:** Schedule of enrollment, interventions, and assessments for study.

Time points		Prescreening telephone call	Baseline assessment T_0_ (<24 days following screening)	Study assessment T_1_ (3-month follow-up)	Study assessment T_2_ (6-month follow-up)
**Enrollment**					
	Explain study	X^a^			
	Screen eligibility criteria	X			
**Outcome measures**					
	SUCCAB^b^ testing		X	X	X
	GDS^c^		X	X	X
	Berkman-Syme		X	X	X
	APHAB^d^		X	X	X
	Randomization after completion of all baseline assessment		X		
**Interventions**					
	Auditory training				
	Hearing aid for Group A participants				
	Hearing aid for Group B participants				

^a^X: Task to be completed.

^b^SUCCAB: Swinburne University Computerized Cognitive Assessment Battery.

^c^GDS: Geriatric Depression Scale.

^d^APHAB: Abbreviated Profile of Hearing Aid Benefit.

### Participant Timeline

**Figure 1:**
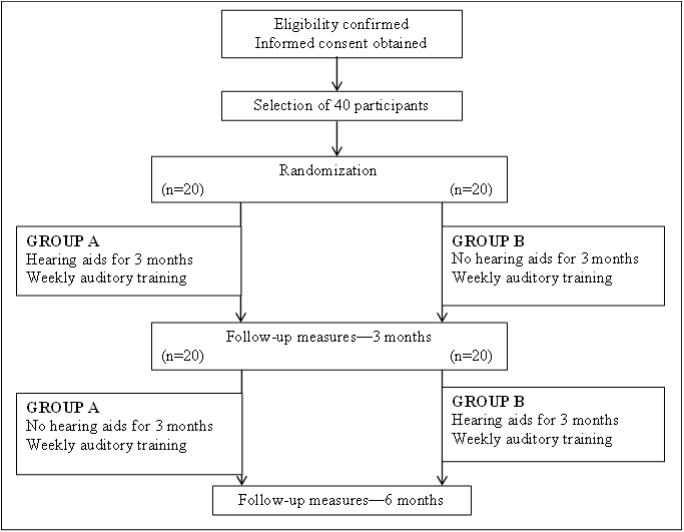
Participant flow diagram.

Participant prescreening and assessment will take place at information sessions that will be held at several independent living aged care facilities located in Melbourne and at Swinburne University of Technology. Independent living aged care facilities with existing relationships with Swinburne University of Technology will be chosen. Participants attending information sessions at Swinburne University will be individuals in the community who have expressed interest in assisting with research projects run by the university, and have therefore provided their contact information to be stored in Swinburne’s Centre for Human Psychopharmacology (CHP) database. After providing informed consent, eligible participants will be randomized into 2 equal groups (A and B) for the study, as described in Figure 1.

### Sample Size

Allowing for 2.5% significance, 80% power, and a moderate effect size (f=0.25), a power analysis indicated that a repeated measures mixed effects design with 3 repeated measures required a total sample size of 34 participants, split evenly between the 2 groups. This allows for the comparison of changes from baseline to 3 and 6 months for the 2 groups. Except for participants from Swinburne’s CHP database who will need to travel to attend their auditory training sessions at Swinburne University, all other participants will attend their appointments at their facilities. As a result, only a 10% allowance was made for attrition resulting in an overall sample size of 40.

### Recruitment

Aged care facility managers will be contacted by telephone to explain the study. If an aged care facility shows interest in the study, researchers will visit the facility to provide the facility manager with more detailed written and oral information. If the facility manager consents for their facility to participate in the study, the study will be advertised at the facility and promotional materials will be distributed to all the residents, inviting them to an information session. Participants from the CHP database will be contacted by the researchers either by telephone or email to explain the study, and participants who express interest will be invited to attend an information session.

At the information session, researchers will explain the purpose and significance of the study. At the same time, a preselection screening will be conducted to identify participants who are willing to wear hearing aids and undergo auditory training to address their hearing loss. Selected participants will be sent a Participant Information and Consent Form package that includes detailed information on the study procedure, a consent form, and a return prepaid envelope. Once written consent is received, participants will be invited to complete baseline measures before enrollment into the study. Recruitment commenced in December 2016.

### Assignment of Interventions

#### Allocation

Groups will be matched in terms of the degree of hearing loss (mild or moderate) with 1 member from each matched pair randomly assigned to *Group A* and the other member of each matched pair assigned to *Group B*. Allocation will be performed using a system of envelopes prepared and opened by the researcher at the time of recruitment.

#### Blinding

Given the nature of the intervention, this study will not be blinded as both investigators and participants will know who is wearing hearing aids in each 3-month period.

### Measures

#### Screening

All enrolled participants will not be cognitively impaired and will be screened for adequate cognitive functioning using MMSE. Participants scoring 24 or lower on MMSE will not be eligible for participation.

#### Swinburne University Computerized Cognitive Assessment Battery

SUCCAB is a validated computer-based cognitive battery consisting of 8 measures that were developed, based on cognitive and neuroimaging literature, to focus on cognitive domains that were most likely to decline with increasing age [45]. Studies using this battery have shown cognitive changes sensitive to interventions in 5 to 16 weeks [47,48]. The SUCCAB battery uses a simple 5-button interface and has been validated in other studies involving the elderly [49,50]. The 8 measures of cognitive tests assessed by SUCCAB consist of Simple and Choice Reaction Times, Immediate and Delayed Recognition, Congruent and Incongruent Stroop color-words, Spatial Working Memory, and Contextual Memory.

A performance score for each task will be calculated as the ratio of accuracy and reaction time. This approach takes into account variations in accuracy and response time because of speed versus accuracy trade-offs in performance.

#### Hearing Assessments

Participants will undergo the following hearing assessments:

##### Otoscopy and Tympanometry

Following otoscopy, all participants will undergo tympanometry and acoustic reflex testing to assess the status of the middle ear.

##### Pure Tone Audiometry in Each Ear

To understand the degree of hearing impairment and classify participants according to the type of hearing loss, hearing ability will be measured at threshold frequencies 0.5, 1, 2, 3, 4 kHz in both ears. The choice of frequency to be tested corresponds to the amplification range of most modern hearing aids and is consistent with capturing sensitivity at frequencies affected by sensorineural hearing loss and noise-induced damage. Only participants with either mild or moderate symmetric sensorineural hearing loss will be included in the study.

##### Blamey Saunders Speech Perception Test

A Web-based Speech Perception Test (SPT) will be used in addition to the standard audiogram for the purpose of measuring hearing loss. SPT is a monosyllabic word test used to characterize the form and degree of hearing loss [23]. There will be 5 SPT evaluations altogether: SPT will be performed without hearing aids at baseline, after 3 months, and then at 6 months for all participants included in the trial. It will also be performed with hearing aids immediately after participants are fitted with hearing aids for the first time and at the end of 3 months of auditory training while wearing a hearing aid.

#### Paper-Based Questionnaire

Participants will complete a paper-based questionnaire, which will be structured in the following sections:

##### Demographics

Information on a variety of demographic variables will be collected to describe the characteristics of the study sample.

##### Geriatric Depression Scale

GDS is a self-rating screening scale for measuring levels of depressive symptoms in elderly population [51]. The short version of GDS will be used [52]. The GDS has been found to be a reliable and valid measure of depressive symptoms [53] and to be highly correlated with other measures of such symptoms. GDS was designed for older adults. Items are scored dichotomously (respondents answer “Yes” or “Not” to 5 items). Items assess nonsomatic aspects of depression, thus allowing for discrimination between respondents with depressive symptom and those with medical problems. A cut-off GDS score of 7 will be used, with a score greater than 7 indicating the presence of depression. Participants will answer GDS at baseline, after 3 months, and then at 6 months.

##### Social Interaction Measure

The Berkman-Syme Social Network Index [54] will be used to assess participants’ social interaction and connections with families and friends. Participants will answer the Berkman-Syme Social Network Index at baseline, after 3 months, and then at 6 months.

##### Abbreviated Profile of Hearing Aid Benefit

APHAB [55] is a self-assessment inventory that will be answered by each participant to assess hearing satisfaction (with or without hearing aids). Participants will answer APHAB at baseline, after 3 months, and after 6 months. Four scales of the APHAB will be assessed, namely, (1) ease of communication, (2) effects of background noise, (3) effects of reverberation, such as listening to sounds across a large room, and (4) aversiveness, which will look at uncomfortable loudness of background sounds such as traffic and alarm bells.

### Statistical Analysis

The following statistical analyses will be performed:

1. Baseline comparison of the 2 groups in terms of demographics, cognition, depression, social interaction, hearing loss, and hearing satisfaction by reporting descriptive statistics of each group as randomized, and an effect size of the difference using Cohen's d.
2. Comparison of the 2 groups in terms of changes in cognition, depression, and social interaction from baseline to 3 months and 6 months, using a per protocol approach for the crossover analysis [39] and an intention-to-treat, multilevel model analysis [56]. These methods will be used to estimate any carryover effects [57].
3. Analysis of SPT results with and without hearing aids as a measure of the efficacy of hearing aids and auditory training with and without hearing aids using multilevel models and again allowing for carryover effects.
4. Analysis of the speech tracking rates from the two 12-week programs of speech tracking using a learning model as described by Blamey and Alcantara [25]. This analysis will yield learning and forgetting rates with and without hearing aids. These learning and forgetting rates are valid measures of cognitive processes that are likely to be affected by the use of hearing aids. These data will also be analyzed using multilevel models again allowing for carryover effects.
5. Analysis of the aided and unaided scores from APHAB will be used to assess how the benefit of hearing aids differed between groups and over degree of hearing impairment (mild/moderate hearing loss).

## Results

This investigation was funded by the Australian Research Council and Blamey and Saunders Hearing Pty Ltd under the Industry Transformation Training Centre Scheme (ARC Project No. IC140100023). The study protocol was reviewed and approved by Swinburne’s Human Research Ethics Committee (SUHREC) on July 22, 2016, protocol number SHR Project 2016/159. The trial is registered in ClinicalTrials.gov with identifier NCT03112850. Recruitment commenced in December 2016 and was completed in June 2017. Researchers obtained written consent from all participants before participating in this trial.

The integrity of the trial, including data collection and monitoring, trial progress, adverse events, and compliance with SUHREC reporting procedures will be overseen by the chief (DM) and associate investigators. No serious adverse events are anticipated. The study coordinator (JN) is responsible for communicating protocol changes to relevant stakeholders, including ClinicalTrials.gov registry.

Data analysis is currently under way and the first results are expected to be submitted for publication in June 2018.

## Discussion

Chronic hearing loss can have a negative impact on several domains of aging such as social engagement, activity, vitality, physical mobility, and cognitive health. Interventions that can significantly delay the onset of sensorineural hearing loss or slow its progression are being actively pursued; however, no disease-modifying treatment is currently available. Understanding the best strategies for aural rehabilitation in older people in whom hearing could compensate for other physical or sensorial limitations may help mitigate cognitive decline.

A limitation of the study is that, it will recruit community-dwelling adults who are not cognitively impaired; hence, they may not show improvement in cognitive functioning because of their high baseline scores. However, by focusing on community-dwelling adults, this research will be able to examine the efficacy of programs aimed at minimizing cognitive decline and reducing the rate of transfer to low- and high-care accommodation.

For the study intervention, auditory training is being used as the comparator rather than hearing aids, which is popularly known as the main clinical management approach for addressing hearing loss. Although this may be a limitation, the concept of auditory training is not new for addressing hearing loss, as its inception can be traced back to the birth of audiology decades ago, when aural rehabilitation programs were first created for people who had suffered hearing loss [58]. Today, auditory training is a common intervention that is effective and is still used in routine practice for pediatric clients who receive rehabilitation services [59] and with clients who receive cochlear implants [60,61]. It is hoped that with individualized face-to-face auditory training as the comparator for this study, participants will be actively involved in the rehabilitation process, leading to increased compliance in terms of hearing aid usage. Auditory training plus hearing aids will also allow us to know whether hearing aids provide any added benefit to face-to-face auditory training.

## Abbreviations

APHAB: Abbreviated Profile of Hearing Aid Benefit
CHP: Centre for Human Psychopharmacology
GDS: Geriatric Depression Scale
LACE: Listening and Communication Enhancement
LOF: Liberty Open-Fit
MMSE: Mini-Mental State Examination
SPT: Speech Perception Test
SUCCAB: Swinburne University Computerized Cognitive Assessment Battery
